# Effects of Birthweight of Piglets in a Multi-Suckling System on Mortality, Growth Rate, Catch-Up Growth, Feed Intake and Behaviour

**DOI:** 10.3390/ani13020297

**Published:** 2023-01-14

**Authors:** Tianyue Tang, Walter J. J. Gerrits, Carola M. C. van der Peet-Schwering, Nicoline M. Soede, Inonge Reimert

**Affiliations:** 1 Animal Nutrition Group, Department of Animal Sciences, Wageningen University & Research, P.O. Box 338, 6700 AH Wageningen, The Netherlands; 2 Adaptation Physiology Group, Department of Animal Sciences, Wageningen University & Research, P.O. Box 338, 6700 AH Wageningen, The Netherlands; 3 Wageningen Livestock Research, Wageningen University & Research, P.O. Box 338, 6700 AH Wageningen, The Netherlands

**Keywords:** group housing, birth weight, body weight gain, piglet survival, homogeneity, behaviour

## Abstract

**Simple Summary:**

Multi-suckling systems aim to improve animal welfare, but in these systems, a large variation is seen in piglet growth rate. This study investigated relationships between birthweight and growth rate, and other piglet traits in this system, and studied if specific traits are indicative of the ability of catch-up growth in low birthweight piglets. We found that birthweight was positively related with survival, growth rate, the number of sucklings, milk intake and feed intake, and with skin lesion scores. Irrespective of birthweight, fast-growing piglets tended to eat more feed, were present less often at teats of alien sows, and had more skin lesions. Our study provides little insight into the piglet traits that affect catchup growth in a multi-suckling environment, but it confirms the effects of piglet birthweight on survival and body weight gain, which is related to increased milk and feed intake.

**Abstract:**

Multi-suckling systems aim to improve animal welfare, but in these systems, a large variation is seen in piglet growth rate. Birthweight (BiW) plays an important role in explaining the variation in body weight (BW) gain of piglets. This study aims to investigate the relationships between BiW and piglet traits up to day 44 postpartum (p.p.). A total of 55 sows were used. The growth rate and mortality were assessed for all piglets. Feed and milk intake, behaviours, and skin lesions were assessed in four focal piglets per litter. Focal piglets were divided into four groups based on their BiW class (high BiW (HBiW) vs. low BiW (LBiW)) and growth rate class (fast vs. slow). Results showed that increased mortality was observed in piglets with a BiW below 1.1 kg. Birthweight was positively related with the number of sucklings (β = 2.8 no./kg of BiW per 7.5 h), corresponding to milk intake (β = 102 g/kg of BiW per day), and to a lesser extent, to the intake of sow feed (β = 44 g/kg of BiW per day) in week 6. Birthweight was positively related with the number of skin lesions (β = 4.3 no./kg of BiW) in week 4. We found no indications that fast-growing LBiW piglets differed from fast-growing HBiW piglets, however, fast-growing piglets of both HBiW and LBiW tended to eat more feed (485 ± 18 vs. 420 ± 17 g/day, *p* = 0.068), were present less often at teats of alien sows (1.1 ± 0.2 vs. 1.8 ± 0.3, *p* = 0.010), and had more skin lesions (9.0 ± 0.6 vs. 7.4 ± 0.4, *p* = 0.047), compared to slow growing piglets. Our study, thus, provides little insight into the traits that affect catchup growth in a multi-suckling environment but increases insight into the differences between fast-growing and slow-growing piglets, regardless of their birthweight class.

## 1. Introduction

Genetic selection for increased litter size during the last decades has coincided with a higher proportion of low birthweight (BiW) piglets within litters, and thus increased within litter variation in BiW [1,2], which consequently results in increased pre-weaning mortality [2,3]. The heterogeneity in weight gradually increases as piglets grow older [4], which negatively affects production efficiency in current all-in-all-out pig production systems [5].

During the last decades, various housing systems have been designed that aim to be closer to the natural living conditions of pigs. One example of such a system is a multi-suckling (MS) system, in which several sows and their litters are housed together during a longer lactation [6,7]. As consequences of body weight (BW) uniformity for subsequent performance have usually been studied in conventional housed pigs; the current study aims to explore this for an MS system.

In conventional housing, variation in BiW was found to account for substantial variation in BW or BW gain of piglets [8,9,10]. For example, BiW was significantly correlated with BW gain during days 0–23 after birth (r = 0.392) [11]. It was found that BiW explained 30–40% of the within-litter variation in BW of piglets at 2 weeks of age [9], 34% of the individual variation in BW of piglets at 21 days of age [12], and 20% of the between-litter variation in BW of piglets at 8 weeks of age [8]. In our previous research in an MS system, we explored factors affecting variation in BW gain of piglets which included BiW, behaviours, genetics, skin lesions and feed intake [13]. We found that BiW, ingestive behaviour, and piglet feed intake were important piglet traits which influenced piglet BW gain variation during lactation. In addition, we found that BiW also played an important role in explaining variation in feed intake during middle lactation in the MS system. In addition to its effect on feed intake, BiW may also play a role in explaining variation in other piglet parameters.

Many studies in conventional housing have shown that BiW affects pre-weaning mortality, as piglets that died before weaning were lighter at birth [2,14,15,16]. For example, Knol et al. (2022) found a curvilinear relationship between individual BiW and survival during lactation, with the highest survival at the highest birthweight; piglets with BiW above 0.9 kg have >70% survival, while piglets with BiW below 0.7 kg have <25% survival [2]. Hales et al. (2013) found that piglets that survived till weaning had a higher BiW than piglets that died before weaning (1.5 vs. 1.2 kg, *p* < 0.001) [16]. As piglet mortality during 0–4 weeks of age, before weaning, was found to be higher in an MS system compared with conventional housing [17], the question arises of how this was related to BiW. Therefore, the first aim of the current study was to investigate the relationships between BiW and piglet traits including growth performance, nutrient intake, behaviours and skin lesions, and to study the relationship between BiW and piglet mortality, up to day 44 of lactation in an MS system.

The majority of the literature suggest an important role of BiW for postnatal growth, with piglets born light more likely to have a lower BW throughout their life [15,18,19]. However, it has been found that some low birthweight piglets are able to show compensatory growth, whereas others are not [4,20]. For example, Douglas et al. (2013) found that piglets with a BiW below the average BiW of the population were able to achieve a subsequent BW that was at or above the average weight of the population for that age [20]. In addition, it was proposed that the impact of BiW on the growth rate of piglets decreases with increasing age [21]. This suggests that BiW is not the sole predictor of postnatal growth performance [22]. In conventional housing, the characteristics that make low birthweight piglets have the ability to compensate for growth have been explored, for example, morphology characteristics such as body mass index and abdominal circumference [22]. Therefore, the second aim of the current study was to investigate piglet traits that can predict whether a piglet has the ability to catch-up or not.

## 2. Materials and Methods

### 2.1. Animals and Experimental Design

Data were utilized and re-analysed from two experiments which were conducted in an MS system, i.e., exp 1 [13] and exp 2 [23], at Swine Innovation Center, Sterksel, The Netherlands.

In total, 55 multiparous sows (Topigs 20) were used in three batches of five sows in exp 1 (parity 3.9 ± 0.4) and eight batches of five sows in exp 2 (parity 3.6 ± 0.2). In each litter, four focal piglets (Tempo × Topigs 20) were selected based on their BiW, which were the piglets with the second lowest and second highest BiW for both the boars and gilts. In exp 2, treatments were applied, having four control (CTRL) batches and four treatment (TREAT) batches. In TREAT of exp 2, interventions were applied aiming at improving the BW gain of small piglets, by later grouping of the piglets at days 13–14 postpartum (p.p.) and split-weaning on day 35 p.p. In both experiments, sows and focal piglets were marked to be individually distinguished.

The MS system consisted of two MS units and one Intermittent Suckling (IS) area [13]. Each MS unit consisted of five farrowing pens connected to a communal MS area including a lying area, a feeding area and a dunging area. Each farrowing pen had a feeding trough with a drinking nipple for the sows, a water nipple for the piglets, and a piglet nest. In the lying and dunging area of the system, three drinking bowls were accessible to both sows and piglets. In the feeding area, there were five feeding places for the sows which were also accessible for piglets, and one piglet feeding area in front of the sow feeding places which was only accessible to the piglets. This piglet feeding area contained three small round feeders (used until day 35 p.p.) containing nine feeding places and a sensor-controlled automatic piglet feeder containing 10 feeding places (used from day 28 p.p.). In exp 2, two extra feed hoppers with sow feed (used from day 21 p.p.) containing four feeding places were provided in the piglet feeding area to enable piglets access to sow feed both during the day and night.

**Figure 1:**
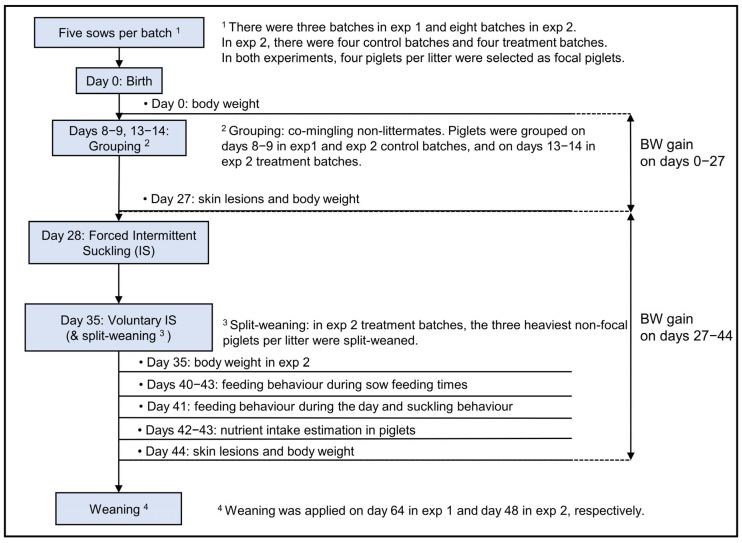
The time schedule of the experimental setup of sows and piglets during lactation in the multi-suckling system.

Per batch, five pregnant sows were introduced into the MS system one week before the expected farrowing date (Figure 1). Sows were restrained between bars in a temporary crate within the farrowing pens from one night before the expected farrowing date until day 3 p.p. to prevent piglet crushing. From day 4 p.p. onwards, the bars were opened to create loose housing. Sows could move freely in and out of the farrowing pens in the days before farrowing and from day 4 p.p. Piglets were given access to the whole system (i.e., grouped) on day 9.1 ± 0.1 p.p. in exp 1 and, dependent on treatment on day 8.2 ± 0.1 p.p. in CTRL and day 13.4 ± 0.1 p.p. in TREAT in exp 2. The average grouping age of exp 1 and 2 was day 10.3 ± 0.3 p.p. On days 28–34 p.p., sows were separated from the piglets for 10 h/day (07:00–17:00) by bringing the sows to the IS area, in which the sows had contact with a boar to stimulate synchronous oestrus. From day 35 p.p. onwards, sows could choose to be in the IS area at all times, whereas piglets could only stay in the MS unit. Piglets were weaned on days 64 and 48 p.p. in exp 1 and 2, respectively. An exception was made for TREAT in exp 2, where the three heaviest non-focal piglets within each litter were weaned and transferred to a rearing department on day 35 p.p. (i.e., split-weaning).

### 2.2. General Housing and Feeding Management

**Figure 2:**
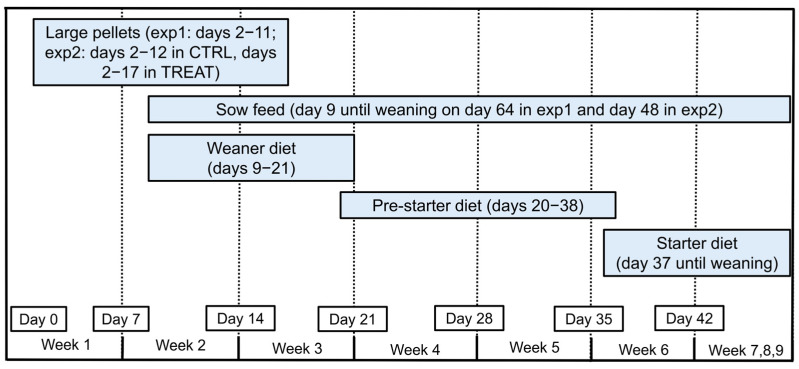
The general feeding management of piglets during lactation in the multi-suckling system.

Piglets were ear tagged within 1 day p.p. Litter sizes were standardized between 24 and 48 h p.p. (exp 1: 13.8 ± 0.3 piglets/litter; exp 2: 14.3 ± 0.1 piglets/litter) according to the number of functional teats available per sow. Piglets were not tail docked, teeth resected or castrated. Piglets firstly received large creep feed pellets (exp 1: from day 2 p.p. until 2 days after grouping; exp 2: from day 2 p.p. until 3 days after grouping), thereafter a weaner diet (on days 9–21 p.p.), pre-starter diet (on days 20–38 p.p.) and starter diet (from days 37 p.p. onwards). In exp 2, TREAT piglets also received sow feed in the farrowing pens on days 9–14 p.p. The general feeding management of piglets is shown in Figure 2.

The sows were fed twice daily at 08:00 and 16:00. From voluntary IS (day 35 p.p.) onwards, in exp 1, sows were fed in the IS area at 08:00 and were floor fed in the MS feeding area at 16:00; in exp 2, sows were fed in the MS feeding area both at 08:00 and 16:00. Water was available ad libitum for sows and piglets. In exp 1 and 2, piglets could eat sow feed together with the sows during sow feeding times; additionally, in exp 2, sow feed was available at all times in sow feed hoppers in the piglet feeding area.

Enrichment materials were provided throughout the lactation period. In the MS units, the enrichment included two hessian sacks in each farrowing pen during farrowing as nesting materials, two handfuls of long straw from day 2 p.p. in each farrowing pen, and five hessian sacks with five ropes, which were hung on the wall in the lying area and were regularly replaced. In the IS area, the enrichment included one rope and two hessian sacks for the sows, and one metal chain and one rope for the boar.

### 2.3. Data Collection from Exp 1 and Exp 2

#### 2.3.1. Body Weight

Body weight data of piglets in both experiments on days 0, 27 and 44 p.p. were used. Body weight data of piglets in exp 2 on day 35 p.p. were additionally used.

#### 2.3.2. Feeding Behaviour during Sow Feeding Times and during the Day

The feeding behaviour of the piglets during sow feeding times was scored live using 2-min instantaneous scan sampling on days 40–43 p.p. at 16:00–16:30 in exp 1, and days 41–43 p.p. at 08:00–08:30 and 16:00–16:30 in exp 2. The feeding behaviour of the piglets during the day was scored live on day 41 p.p. at 08:30–16:00, using 30-min and 15-min instantaneous scan sampling in exp 1 and 2, respectively.

During these observation periods, for each focal piglet, it was noted whether a piglet was in the feeding area, contacting (i.e., sniffing or eating) sow feed or contacting (i.e., sniffing or eating) piglet feed. From these observations, the percentage of time spent on contacting sow feed and piglet feed was calculated per piglet.

#### 2.3.3. Suckling Behaviour

In both experiments on day 41 p.p. at 08:30–16:00, the frequency of presence at teats of each focal piglet in all successful suckling bouts (at biological mother and other sows), on either the front (the first two pairs of teats), the rear (the last two pairs of teats) or the middle teats (the remaining teats) was determined. An unsuccessful nursing bout was defined when it started within 20 min after a previous nursing bout [24] and with no milk let-down. The unsuccessful bouts were subsequently excluded from the analysis. The frequency of presence at teats at both their own mother and cross-suckling sows was calculated per piglet during the 7.5 h of observations; the frequency of presence at alien teats, i.e., the teats of cross-suckling sows was calculated per piglet during the 7.5 h of observations as well. The frequency of presence at the front and middle teats were summed into one variable for further analysis.

#### 2.3.4. Skin Lesions

On day 44 p.p., the number of skin lesions was counted per piglet by visual assessment as the number of fresh lesions on the whole body, except for ears and tails. Scoring was performed according to [13]. Skin lesions can be regarded as a proxy for aggressive behaviour given and received [25].

#### 2.3.5. Individual Nutrient Intake

The dual marker method [26] was used to measure individual dry matter (DM) intake of sow feed and piglet feed on days 42–43 p.p. The dual marker included two types of markers, i.e., a reference marker and an in-feed marker. N-alkanes C31 and C36 were considered as in-feed markers for the sow and piglet diets, respectively, with a concentration of 40–50 mg/kg C31 in the sows’ diets and 160–170 mg/kg C36 in the piglets’ diets. The alkane C31 was provided via the inclusion of 15% alfalfa in the sow feed. The alkane C36 was melted on soybean meal in a forced air oven followed by mixing it into the piglet feed. The reference marker C32 was provided to the piglets on days 42–43 p.p. via a feed bolus for 3 times/day at 08:30, 14:30 and 20:30, with each bolus (~2.0 g) containing 20 mg of C32. On day 44 p.p., two spot faecal samples were collected from each focal piglet at 08:30 and 12:30. N-alkanes in faecal and feed samples were measured by gas chromatography [27].

DM intake of sow feed and piglet feed in each piglet was calculated for days 42–43 p.p. using Equation (1):

(1) $$\mathrm{Estimated}\;\mathrm{intake}\;\mathrm{of}\;\mathrm{piglet}\;\mathrm{or}\;\mathrm{sow}\;\mathrm{feed}\;(g/\mathrm{day})\;=\frac{\mathrm{concentration}\;\mathrm{of}\;\mathrm{in}-\mathrm{feed}\;\mathrm{marker}\;\mathrm{in}\;\mathrm{faeces}\;(\mathrm{mg}/\mathrm{kg})}{\;\mathrm{concentration}\;\mathrm{of}\;\mathrm{reference}\;\mathrm{marker}\;C32\;\mathrm{in}\;\mathrm{faeces}\;(\mathrm{mg}/\mathrm{kg})}\;\text{×}\;\mathrm{daily}\;\mathrm{intake}\;\mathrm{of}\;\mathrm{reference}\;\mathrm{marker}\;C32\;(\mathrm{mg}/\mathrm{day})/\mathrm{concentration}\;\mathrm{of}\;\mathrm{in}-\mathrm{feed}\;\mathrm{marker}\;\mathrm{in}\;\mathrm{diet}\;(\mathrm{mg}/\mathrm{kg})\;\text{×}1000$$

Milk intake was calculated using Equation (2), assuming fixed feed conversion ratios (FCR) of converting DM feed intake into BW gain of 1.5 g/g, and assuming a fixed efficiency of converting fresh milk into BW gain of 4.89 g/g [28].

(2) Estimated intake of milk (g/day)=(BW gain (g/day) – intake of total feed (g/day)/FCR (g/g)) × 4.89

DM intake of milk was then calculated assuming a DM content of 19% [29]. The complete procedures for the calculation of nutrient intake can be found in [13]. For BW gain in Equation (2), in exp 1, BW gain was calculated on days 27–44 p.p., while in exp 2 BW gain was calculated on days 35–44 p.p.

#### 2.3.6. Piglet Mortality

For all piglets in the MS system, the percentage of piglet mortality per litter (%) and the percentage of crushed piglets per litter (%) on days 0–8 p.p., days 0–26 p.p. and on days 0–44 p.p. were calculated. The number of dead and crushed piglets per litter were also calculated. Stillborn piglets were excluded.

All piglets in the MS system were then grouped into 14 BiW categories, ranging from 0.7 to 2.0 kg in 0.1 kg intervals. Piglets with BiW below 0.7 and over 2.0 kg were placed into the 0.7 and 2.0 kg BiW class, respectively. There were 14 and 9 piglets born with BiW below 0.7 and over 2.0 kg, respectively. Piglet mortality (dead piglets/total number of alive piglets on day 0 p.p. × 100%) within each BiW class was calculated for two periods, i.e., days 0–26 and 27–44 p.p. Stillborn piglets were excluded.

### 2.4. The Definition of Fast and Slow-Growing Piglets

Focal piglets were divided into four groups based on their BiW class and growth rate class. BiW class: high BiW (HBiW) focal piglets and low BiW (LBiW) focal piglets. Growth rate class (fast vs. slow): Within each BiW class, focal piglets were defined as fast-growing when their BW on day 44 p.p. was equal to or exceeded the median BW of their litter; other focal piglets were identified as slow growing. LBiW-fast growing piglet was also called a catch-up low BiW piglet.

### 2.5. Statistics

Statistical analyses were conducted with SAS 9.4. Data were merged from exp 1 and 2. The variables of DM milk intake (g/day) and contacting piglet feed during the day (% of observations) were the residuals of the original values corrected for treatment effect in exp 2, as these were the only variables significantly affected by treatment among all measured variables in exp 2.

The effect of sex (boars vs. gilts) and birthweight (continuous) and their interaction on multiple response variables including BW gain, nutrient intake, feeding and suckling behaviour and skin lesions were analysed by analysis of covariance using the General Linear Models (GLM) procedure. Experiment was also included in the GLM models as a fixed effect. The interaction between experiment and sex, and the interaction between experiment and birthweight were initially included in the GLM models as well, but were removed from the final models as these did not reach statistical significance (*p* > 0.05).

The effects of BiW class (HBiW vs. LBiW), growth rate class (fast vs. slow) and their interaction on multiple response variables were analysed by analysis of variance. Experiment was also included in the models as a fixed effect. The interaction between experiment and BiW class, and the interaction between experiment and growth rate class were initially included in the models as well, but were removed from the final models as these did not reach statistical significance (*p* > 0.05). Batch nested within experiment was included as random effect. When the interaction between BiW class and growth rate class was significant, it was further investigated with post hoc pairwise comparisons using the differences of the least squares means among four types of focal piglets (HBiW-fast, HBiW-slow, LBiW-fast, LBiW-slow). For continuous response variables, i.e., BW, BW gain, and DM intake of feed and milk, the normality of model residuals was checked using PROC UNIVARIATE. The distribution of residuals in the model with DM intake of milk as a response variable was not normal; therefore, DM milk intake was converted using log (1 + N) before analysis in PROC MIXED. For the other continuous variables, the distribution of residuals was normal and PROC MIXED was used. For proportional response variables, i.e., the proportion of time spent on contacting sow feed and piglet feed during sow feeding times and during the day which was in the range of 0–1, PROC GLIMMIX with a beta distribution and logit link function was used; when the proportion was equal to 0 and 1, it was converted to 0.0000001 and 0.9999999 before analysis, respectively, to accommodate a beta distribution. For count response variables, i.e., the frequency of presence at teats and skin lesions, PROC GLIMMIX with Laplace approximation, Poisson distribution and log link function were initially used. In models where no evidence of overdispersion was present, i.e., the values of Pearson Chi-Square/DF were smaller than one [30], Poisson distribution was used; When overdispersion was detected, a negative binomial distribution was used as an alternative for the Poisson distribution. Statistical significance was set at *p* < 0.05 and tendency was set at 0.05 < *p* < 0.10. Data are presented as mean ± SEM.

## 3. Results

### 3.1. Birthweight and Mortality

The percentage of piglet mortality per litter (%) and the percentage of crushed piglets per litter (%) during days 0–8 p.p. were 14.4 ± 1.8% and 9.9 ± 1.4%, respectively; during days 0–26 p.p. were 20.5 ± 2.2% and 14.5 ± 1.8%, respectively; during days 0–44 p.p. were 21.6 ± 2.2% and 14.9 ± 1.9%, respectively. The number of dead and crushed piglets per litter during days 0–8 p.p. were 2.3 ± 0.3 and 1.6 ± 0.2, respectively; during days 0–26 p.p. were 3.2 ± 0.4 and 2.3 ± 0.3, respectively; during days 0–44 p.p. were 3.4 ± 0.4 and 2.3 ± 0.3, respectively.

**Figure 3:**
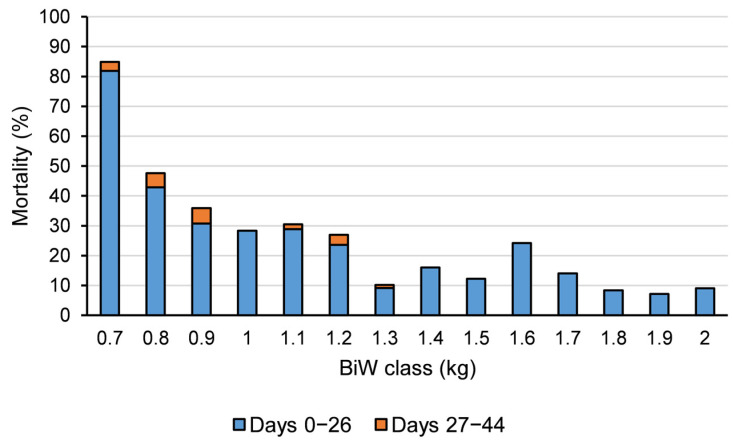
Piglet mortality (%) in different birthweight (BiW) classes on days 0–26 postpartum (p.p.) and 27–44 p.p. in all piglets up to day 44 of lactation in the multi-suckling (MS) system. Piglet mortality was calculated as the number of dead piglets divided by the number of both dead and alive piglets within BiW class. Piglets with BiW <0.7 and >2.0 kg are included in the 0.7 and 2.0 kg BiW class, respectively. Stillborn piglets were excluded from the data set and piglets which were cross-fostered in the MS system were included in the data set. Data were merged from two animal experiments conducted in the MS system. In exp 1, piglets were grouped on days 8–9 p.p. In exp 2, piglets in control groups were grouped on days 8–9 p.p. and no split-weaning was applied; piglets in treatment groups were grouped on days 13–14 p.p. and the three heaviest non-focal piglets per litter were split-weaned on day 35 p.p. Previously we found no treatment effect on most of the measured variables of piglets in exp 2.

Number of dead and alive piglets in different BiW classes on days 0–26, days 27–44 up to day 44 of lactation was summarized in Figure S1. As shown in Figure 3, there was a curvilinear relationship between piglet mortality (%) and BiW, where mortality was higher for low BiW piglets. Mortality on days 0–26 p.p. and 0–44 p.p. declined and plateaued when BiW increased above 1.0 kg. Of all piglets that died during days 0–44 p.p., 68.3% of the piglets died during days 0–8 p.p.; 68.5% of the piglets died during days 0–8 p.p. was due to crushing. 68.8% of the piglets died during days 0–44 p.p. was due to crushing.

### 3.2. Birthweight and Piglet Traits

As shown in Table 1, in all piglets, BiW (kg) was positively related to BW gain (g/day) during days 0–27 p.p. (β_BiW_ = 102 g/kg of BiW per day, *p* < 0.001), 27–44 p.p. (β_BiW_ = 110 g/kg of BiW per day, *p* < 0.001) and 0–44 p.p. (β_BiW_ = 102 g/kg of BiW per day, *p* < 0.001). Likewise, in focal piglets, BiW was positively related to BW gain on days 0–27 p.p. (β_BiW_ = 85 g/kg of BiW per day, *p* < 0.001), 27–44 p.p. (β_BiW_ = 103 g/kg of BiW per day, *p* < 0.001) and 0–44 p.p. (β_BiW_ = 91 g/kg of BiW per day, *p* < 0.001), and this relation was similar for both sexes.

Other parameters were available for focal piglets only. During days 42–43 p.p., BiW tended to be positively related to DM intake of sow feed (β_BiW_ = 44 g/kg of BiW per day, *p* = 0.065) and milk intake (β_BiW_ = 102 g/kg of BiW per day, *p* < 0.001). Birthweight was not related to DM intake of piglet feed, DM intake of total feed, the percentage of time spent on contacting feed during sow feeding times on days 40–43 p.p., or contacting total feed during the day on day 41 p.p. Both the percentage of time spent on contacting sow feed during the day and piglet feed during the day on day 41 p.p. were affected by BiW in gilts (β_BiW_ = 4.6%/kg of BiW, *p* = 0.038 and β_BiW_ = −3.4%/kg of BiW, *p* = 0.081, respectively) (interaction effect: *p* = 0.041 and *p* =0.055, respectively), but not in boars. Birthweight was positively related to the frequency of presence at the front and middle teats (β_BiW_ = 2.3 no./kg of BiW per 7.5 h, *p* = 0.009) and total teats on day 41 p.p. (β_BiW_ = 2.8 no./kg of BiW per 7.5 h, *p* < 0.001), but it was not related with the frequency of presence at rear teats or alien teats. Birthweight was positively related with skin lesions on day 27 p.p. (β_BiW_ = 4.3 no./kg of BiW, *p* = 0.012).

### 3.3. The Effect of Growth Rate Class and Birthweight Class on Piglet Traits

Table 2 shows the piglet traits as affected by BiW class (high BiW vs. low BiW) and the growth rate class (fast vs. slow). For BW and BW gain, the interaction between BiW class and growth rate class was not significant (Table 2). BiW class only significantly affected BW on day 27 p.p., with HBiW piglets having a higher BW on day 27 p.p. than LBiW piglets (7.7 ± 0.1 vs. 6.8 ± 0.1 kg, *p* = 0.016). BiW class did not affect BW on day 44 p.p., nor BW gain on days 0–27, 27–44 or 0–44 p.p.

None of the nutrient intake parameters were different among the four types of piglets. For feeding behaviours, the interaction between BiW class and growth rate class tended to be significant only for the variable contacting piglet feed during sow feeding times on days 40–43 p.p. (*p* = 0.071), where HBiW-fast piglets tended to spend more time on contacting piglet feed during sow feeding times than LBiW-fast piglets (7.5 ± 0.8 vs. 6.6 ± 1.1%, *p* = 0.065). Suckling behaviours, i.e., the frequency of presence at teats were not different among the four types of piglets. For skin lesions, the interaction between BiW class and growth rate class tended to be significant on day 27 p.p. (*p* = 0.084), where within HBiW piglets, HBiW-fast piglets had more skin lesions than HBiW-slow piglets (5.7 ± 0.7, vs. 3.9 ± 1.2, *p* = 0.010), while this difference did not exist within LBiW piglets.

For the effect of growth rate class, fast-growing piglets from both the high and low BiW classes on average had a higher BW on day 27 p.p. (7.8 ± 0.1 vs. 6.6 ± 0.1, *p* < 0.001), and day 44 p.p. (15.4 ± 0.2 vs. 12.8 ± 0.2, *p* < 0.001), and a higher BW gain on days 0–27 p.p. (233 ± 4, vs. 195 ± 5, *p* < 0.001), days 27–44 p.p. (448 ± 8 vs. 367 ± 8, *p* < 0.001), and days 0–44 (316 ± 4, vs. 261 ± 5, *p* < 0.001), compared to slow-growing piglets. Correspondingly, fast-growing piglets tended to have a higher total feed intake on days 42–43 p.p. (485 ± 18 vs. 420 ± 17, *p* = 0.068). They tended to spend less time on contacting sow feed during the day on day 41 p.p. (4.5 ± 0.5 vs. 4.9 ± 0.5, *p* = 0.095). Fast-growing piglets were present less often at alien teats on day 41 p.p. (1.1 ± 0.2 vs. 1.8 ± 0.3, *p* = 0.010), and had more skin lesions on day 44 p.p. (9.0 ± 0.6 vs. 7.4 ± 0.4, *p* = 0.047) than slow-growing piglets.

## 4. Discussion

The first aim of the current study was to investigate the relationships between BiW and several piglet traits including nutrient intake, feeding and suckling behaviours and skin lesions, which have been shown to influence the variation in BW gain of piglets in an MS system [13]. In addition, we studied the relationship between BiW and piglet mortality, up to day 44 of lactation. We found increased mortality during lactation in piglets with a birthweight of less than 1.1 kg. Birthweight was positively related with the number of sucklings, correspondingly to milk intake, and to a lesser extent, to the intake of sow feed. In addition, it was positively related with skin lesions. As some low birthweight piglets seem to have the ability to compensate their growth while some cannot, the second aim of the current study was to investigate what characteristics affect the ability of low birthweight piglets to catchup. We found no indications that fast-growing, LBiW piglets differ from fast-growing, HBiW piglets. However, our study increases insight into the way that fast-growing piglets differ from slow-growing piglets, regardless of their BiW class. For piglets born both small and big, fast-growing piglets tended to eat more feed, were present less often at teats of alien sows and had more skin lesions, compared to slow-growing piglets.

### 4.1. Birthweight and Mortality

In the current study, we found that the percentage of piglet mortality per litter (%) during days 0–8 p.p. and 0–44 p.p. were 14.4% and 21.6%, respectively. It was a bit lower than the mortality in the study of [31], who found 17% (days 0–7) and 27% (days 0–44) in an MS system where litters were grouped after week 1 p.p. In the current study, the number of dead piglets per litter during days 0–8 p.p. and 0–26 p.p. was 2.3 and 3.2, respectively, which was comparable with observations in the MS system by [17]. The high mortality in the MS system compared to conventional housing (10–15%) [32,33,34] was also observed by [35] and is likely caused by the increased crushing in farrowing pens before grouping [17,35].

In the current study, we found that the majority (68.3%) of the piglets dying occurred during days 0–8 p.p. which is in agreement with [31]; and of these, the majority (68.5%) was due to crushing, which was in agreement with observations by [17]. Furthermore, in conventional housing, it was reported that during lactation on days 0–25 p.p., more than half of the mortality (57.3%) occurred during days 0–4 p.p. and the majority of these were due to crushing (67.4%) [36].

In the current study, we observed higher mortality of low BiW piglets. Similarly, several studies both in conventional housing and loose farrowing found that piglet mortality during lactation was negatively related with BiW [11,16,37]. Furthermore, we found that the relationship between total piglet mortality up to day 44 p.p. and BiW is curvilinear, where the mortality rate declined and plateaued when BiW increased above 1.0 kg. Knol et al. (2022) also found a curvilinear relationship between individual BiW and lactation survival, with the survival increased and plateaued above 1.2 kg [2]. Marchant et al. (2000) found a curvilinear relationship between BiW and survival rate as well, and they found that only 28% of piglets weighing less than 1.1 kg at birth survived after 7 days [36]. It was shown that piglets weighing less than 1.1 kg are particularly at risk in a cold environment [38,39]. It could be that low BiW piglets have a reduced thermoregulation ability [39,40] due to a greater surface-to-body mass ratio [41] and are less able to compete for teats during suckling. Therefore, these low birthweight piglets stay longer close to the sow to minimize heat loss [39] and spend more time near the sow for stimulating the udder [42,43], which increases the risk of being crushed by the sow. It was found that a large proportion of crushed piglets had empty stomachs [16,44]. For example, 48% and 21% of crushed piglets were found to have no milk in their stomachs which died on days 0–1 and days 2–26 p.p., respectively [16].

In summary, piglets with a BiW of less than 1.1 kg have an increased risk for mortality during lactation. Of all the dead piglets on days 0–44 p.p., the majority of them dying on days 0–8 p.p. was due to crushing. To reduce piglet mortality in the MS system, further interventions before grouping should be considered, including reducing crushing before grouping and ensuring enough heat protection and milk intake for especially piglets with lower BiW.

### 4.2. Birthweight and Piglet Traits

For the piglets that survived till day 44 p.p. in the current study, BiW was positively related with BW gain on days 0–27 p.p., 27–44 p.p. and the total period on days 0–44 p.p. Similarly, in conventional housing, it was found that BiW was significantly correlated with BW gain during days 0–23 p.p. pre-weaning (r = 0.392) [11], and was significantly related with BW at the end of the nursery phase at week 10 p.p. [4].

We investigated the underlying relations of BiW and BW gain by investigating several piglet traits, i.e., intake of nutrients and behavioural characteristics and we found that both the intake of sow feed, the intake of milk, and the level of skin lesions were related to BiW. For intake of sow feed in week 6, we found that a 100 g increase in BiW was related with a 4.4 g/day increase in intake of sow feed and a numerical increase of total feed intake of 5.0 g/day. The relatively high contribution of sow feed intake could be related to the floor feeding of the sows, possibly enhancing the intake of sow feed of piglets by maternal learning [45]. This effect of maternal learning might be more obvious on piglets with higher BiW, as they are stronger and might attack smaller piglets to get away from the sow feeding area. Similar to the general relationship between intake of solid feed and BiW in the current study, Van der Peet-Schwering et al. (2013) also found that piglets with a high BiW (averaged 1.56 kg) ate more from weaning up to 5 weeks after weaning in conventional housing, compared to piglets with a low BiW (averaged 1.13 kg) (feed intake: 0.66 vs. 0.60 kg/day per pig, *p* < 0.001) [46]. Piglets with a higher BiW have a higher number of muscle fibres at birth [47] which may result in higher muscle accretion and higher feed intake [48]. It might also be that piglets with a higher BiW have a better developed digestive system which allows them to ingest more feed [49].

For milk intake measured in week 6, we found that a 100 g increase in BiW was related with a 10 g/day increase in DM milk intake; BiW was also positively related with the frequency of presence at sucklings and specifically at the front and middle teats, measured in week 6. It was proposed in conventional housing that the competitive disadvantage for teats of the smaller piglets compared to the large ones remains throughout lactation [44]. The lower competence of piglets with lower BiW might make them miss more suckling bouts than other piglets, which may explain the positive relationship between BiW and the frequency of presence at sucklings. Front and middle teats were shown to produce higher quantities of milk [50,51,52] and higher concentrations of immunoglobulin in colostrum [53] than do rear teats. For example, the front and middle teats produced 41 g milk/suckling during weeks 1–4 p.p., while the rear teats produced 31 g milk/suckling [52]. Possibly, piglets with higher BiW are more successful in competing for more productive teats, thus having an advantage in obtaining nutritional and immunological components. As a result, they may grow faster and healthier than piglets with lower BiW which suckle the rear teats throughout lactation. It was shown that the degree of mammary development during lactation is dependent on the extent of suckling intensity by piglets [54]. As sow grunting appears to attract piglets to the front teats [52] and piglets with higher BiW tend to win more fights and occupy the front teats [55], these piglets may have more intensive massage towards the front teats, hence stimulating higher milk yield of the front teats [56].

We found that a 100 g increase in BiW was related with an increase of 0.4 skin lesions counted on day 27 p.p. The scoring of skin lesions has been used as an indicator of aggressive behaviour given and received [25,57]. Skin lesions could be caused by the reciprocal fighting activity among unfamiliar non-littermates immediately after grouping [58] and competitive aggressive behaviour towards food [59]. As grouping has been applied for 14–19 days until day 27 p.p., the skin lesions on day 27 p.p. are more possibly caused by competitive behaviour for food. In exp 1 and 2, on day 27 p.p., the piglet: feeding place ratio was 7:1 and 4:1, respectively, which probably provides a competitive environment for piglets. In addition, as the body size of piglets becomes bigger with increased age, the competition among piglets towards teats might also be bigger with increased age until day 27 p.p. It might be that the increased nutrient requirements of high BiW piglets cause them to fight for nutrient resources both in the feeding area and around the teats during suckling bouts, thus having more skin lesions on day 27 p.p.

In summary, we confirm the previously published link between BiW and BW gain of piglets and provide insight into the traits that are affected by BiW throughout lactation in an MS system. We found that BiW was positively related with the number of sucklings, correspondingly to milk intake, and to a lesser extent to the intake of sow feed. In addition, BiW was positively related with skin lesions.

### 4.3. The Effect of Growth Rate Class and Birthweight Class on Piglet Traits

As discussed above, BiW was positively related with subsequent growth performance throughout lactation in the MS system. Previously, low BiW piglets have been shown capable of showing catch-up growth [4,20]. We, therefore, investigated which pig traits affected piglets with low BiW to become a catchup-piglet. We divided all focal piglets into four groups, with the combination of BiW class (high vs. low) and growth rate class (fast vs. slow); therein, fast and slow-growing piglets were defined within each BiW class when their BW on day 44 p.p. exceed or did not exceed the median value of their litter. Interactions between BiW class (high vs. low) and growth rate class (fast vs. slow growing) would then be indicative for catchup piglets in the current data. We found that the interaction between BiW class (high vs. low) and growth rate class (fast vs. slow) was only present for the piglet traits contacting piglet feed during sow feeding times and skin lesions. For most of the piglet traits, including BW gain, feed and milk intake, feeding behaviour, suckling behaviour and skin lesions, neither the interaction between BiW class (high vs. low) and growth rate class (fast vs. slow), nor the effect of BiW class alone was present, indicating that the catchup growth effects were similar for high and low BiW piglets. Effects of growth rate class existed on some piglet traits, i.e., BW gain on days 0–27, 27–44 and 0–44 p.p., total feed intake, the frequency of presence at teats of alien sows and skin lesions. Therefore, our study demonstrated that the majority of the piglet traits measured are not specifically affecting LBiW piglets; instead, for piglets born both small and big, fast-growing piglets ate more feed, were present less often at alien teats and had more skin lesions.

In the current study, we found that the interaction between BiW class and growth rate class was only present for the traits contacting piglet feed during sow feeding times and skin lesions. HBiW-fast piglets tended to spend more time on contacting piglet feed during sow feeding times than LBiW-fast piglets, and HBiW-fast piglets had more skin lesions than HBiW-slow piglets. However, for these two traits, there were no differences between LBiW-fast and LBiW-slow piglets, and the reason is not clear.

The effect of growth rate class existed on some piglet traits, i.e., BW gain, feed intake, contacting sow feed during the day, skin lesions and the frequency of presence at teats of alien sows. In the current study, fast-growing piglets had a higher total feed intake than slow-growing piglets in week 6. Similarly, other studies also reported that feed intake plays an important role in the growth rate of piglets after week 4 [6,60]. In the current study, fast-growing piglets had more skin lesions than slow-growing piglets in week 6. Similarly, Turner et al. (2006) also found a positive correlation between BW and lesion scores in post-weaned pigs after week 4 [25]. It was reported that in socially stable groups, the majority of aggressive behaviours occur around the feeding area to obtain limited food resources [61]. In the current study, the limited piglet: feeding place ratio in week 6 (6:1 in exp 1 and 4:1 in exp 2) might provide a competitive environment for piglets, especially during the synchronization period when groups of piglets are eating at the same time in the piglet feeding area [62]. It might be that in week 6 these fast-growing piglets tend to have a higher motivation to feed [63], have more competition at the feeder [63,64], and are therefore involved in more mutual aggressions with other piglets, leading to skin lesions [65].

In the current study, fast-growing piglets tended to spend less time on contacting sow feed during the day than slow-growing piglets. However, as previously mentioned, we found a positive relationship between BiW and intake of sow feed, the reason for which is not clear. It indicates that the growth rate does not correspond to BiW, and the percentage of time spent on contacting sow feed during the day does not correspond to the intake of sow feed. For the latter, it might be that feeding behaviour alone does not accurately reflect feed intake in late lactation [13], as generally the ingestion frequency decreased and intake per meal increased with the increased age of piglets [66].

In the current study, fast-growing piglets were present less often at the teats of alien sows than slow-growing piglets. It was reported that piglets tend to become a cross-suckler when it has to compensate for the low milk yield of their own mother by cross-suckling an alien sow with a higher milk yield [67]. It could be that due to the better ability to compete for teats, fast-growing piglets are able to fulfil their need for milk already from their own mother, thus were present less often at teats of alien sows. After starting IS, the slow-growing piglets, especially when their own mother was in the IS area, or had a low milk yield, are more likely to become cross-sucklers. However, as the milk yield of sows reaches the peak in week 4 and decreases afterwards [68], these cross-sucklers probably could not even get enough milk from alien sows in week 6 thus leading to a reduced growth rate. It was previously reported that the IS strategy increased the dependency of the piglets on solid feed [69]. Therefore, it could also be that fast-growing piglets might have a higher dependency on solid feed than slow-growing piglets due to the more mature digestive system, thus they might have less possibility to become cross-sucklers in week 6.

## 5. Conclusions

The current study confirms previously published links between birthweight and survival, and the links between birthweight and BW gain of piglets and provides insight into the traits that are affected throughout lactation in an MS system. Increased mortality was observed in piglets with a BiW below 1.1 kg. Birthweight was positively related with the number of sucklings, correspondingly to milk intake, and to a lesser extent to the intake of sow feed. In addition, it was positively related with skin lesions. We found no indications that fast-growing, LBiW piglets differed from fast-growing, HBiW piglets and hence, our study provides little insight into the mechanisms of catchup growth in an MS environment. However, our study increases insight into the way that fast-growing piglets differ from slow-growing piglets, regardless of their BiW class. For piglets born both small and big, fast-growing piglets tended to eat more feed, were present less often at teats of alien sows and had more skin lesions, compared to slow-growing piglets.

## Figures and Tables

**Table 1:** Results of the linear regression analysis between response variables and birthweight (BiW, kg) in all piglets and focal piglets ^1^ up to day 44 of lactation in the multi-suckling (MS) system.

		Mean ± SEM	β_BiW_ ^2^	*p* ^3^	
				BiW	Sex
Response variables ^4^ for all piglets (*n* = 621 on day 44)					
BW gain (g/day)	Days 0–27	214 ± 2	102	**<0.001**	0.153
	Days 27–44 ^5^	398 ± 4	110	**<0.001**	**0.024**
	Days 0–44 ^6^	285 ± 2	102	**<0.001**	**0.027**
Response variables for focal piglets (*n* = 214 on day 44)					
BW gain (g/day)	Days 0–27	216 ± 3	85	**<0.001**	0.270
	Days 27–44	412 ± 6	103	**<0.001**	0.904
	Days 0–44	291 ± 4	91	**<0.001**	0.549
Nutrient dry matter (DM) intake (g/day) on days 42–43	Sow feed	163 ± 6	44	**0.065**	0.793
	Piglet feed	293 ± 12	5	0.918	0.832
	Total feed	456 ± 13	50	0.354	0.925
	Milk	137 ± 7	102	**<0.001**	0.420
Feeding behaviour during sow feeding times (%) on days 40–43	Contacting sow feed	11.9 ± 0.7	1.3	0.609	0.221
	Contacting piglet feed	7.1 ± 0.5	2.7	0.205	0.832
	Contacting total feed	18.9 ± 0.8	4.0	0.235	0.410
Feeding behaviour during the day (%) on day 41	Contacting sow feed ^7^	4.7 ± 0.3	---	0.278	**0.035**
	Contacting piglet feed ^8^	4.6 ± 0.3	---	0.481	**0.045**
	Contacting total feed	9.3 ± 0.5	0.8	0.672	0.968
Suckling behaviour: the presence at teats (no./7.5 h) on day 41	Front and middle teats	4.3 ± 0.2	2.3	**0.009**	0.883
	Rear teats	1.7 ± 0.2	0.5	0.524	0.898
	Total teats	6.0 ± 0.2	2.8	**<0.001**	0.746
	Alien teats	1.4 ± 0.2	−0.3	0.705	0.467
Skin lesions (no.)	Day 27	5.2 ± 0.4	4.3	**0.012**	0.105
	Day 44	8.3 ± 0.5	3.3	0.108	0.746

^1^ Focal piglets: On day 14 postpartum (p.p.), in each litter, the surviving second highest and lowest BiW piglets from both sexes were selected as focal piglets. ^2^ β_BiW_: the regression coefficient of BiW, which was averaged over both sexes. ^3^ *p*-values less than 0.100 are presented in bold. ^4^ The explanation of measured variables is further explained in the text in Section 2.3. ^5^ Body weight (BW) gain of all piglets on days 27–44: least squares means ± SEM: boars: 379 ± 5 g/day, gilts: 394 ± 5 g/day. ^6^ BW gain of all piglets on days 0–44: least squares means ± SEM: boars: 276 ± 3 g/day, gilts: 285 ± 3 g/day. ^7^ Contacting sow feed during the day: *p* (sex × BiW interaction) = 0.041; Boars: Mean ± SEM: 4.9 ± 0.5 (%), β_BiW_ = −1.4, *p* = 0.466; Gilts: Mean ± SEM: 4.4 ± 0.5 (%), β_BiW_ = 4.6, *p* = 0.038. ^8^ Contacting piglet feed during the day: *p* (sex × BiW interaction) = 0.055; Boars: Mean ± SEM: 4.4 ± 0.4 (%), β_BiW_ = 1.6, *p* = 0.357; Gilts: Mean ± SEM: 4.9 ± 0.4 (%), β_BiW_ = −3.4, *p* = 0.081.

**Table 2:** Body weight (BW), BW gain, nutrient intake, feeding behaviours, suckling behaviours, skin lesions, and genetics in four types of focal piglets ^1^ i.e., HBiW-fast, HBiW-slow, LBiW-fast and LBiW-slow up to day 44 of lactation in the multi-suckling (MS) system.

	Mean ± SEM				*p* ^3^		
	Piglet Type ^2^						
Variables ^4^	HBiW-Fast	HBiW-Slow	LBiW-Fast	LBiW-Slow	BiW Class ^2^	Growth Rate Class ^2^	Interaction
No. of alive piglets on day 44							
	89	18	29	78	---	---	---
BW (kg)							
Day 0 ^5^	1.6 ± 0.0	1.7 ± 0.0	1.3 ± 0.0	1.3 ± 0.0	---	---	---
Day 27	7.9 ± 0.1	7.1 ± 0.3	7.5 ± 0.2	6.5 ± 0.1	**0.016**	**<0.001**	0.579
Day 44	15.6 ± 0.2	12.9 ± 0.4	14.8 ± 0.4	12.8 ± 0.2	0.151	**<0.001**	0.693
BW gain (g/day)							
Days 0–27	234 ± 4	200 ± 12	228 ± 8	194 ± 5	0.416	**<0.001**	0.868
Days 27–44	453 ± 9	353 ± 16	431 ± 17	371 ± 9	0.609	**<0.001**	0.529
Days 0–44	319 ± 5	255 ± 8	307 ± 9	262 ± 5	0.604	**<0.001**	0.537
Nutrient dry matter (DM) intake (g/day) on days 42–43							
Sow feed	171 ± 9	159 ± 25	175 ± 16	150 ± 9	0.800	0.157	0.382
Piglet feed	326 ± 21	252 ± 38	275 ± 32	272 ± 19	0.461	0.198	0.497
Total feed	497 ± 21	411 ± 33	450 ± 34	423 ± 20	0.436	**0.068**	0.764
Milk ^6^	165 ± 14	87 ± 14	145 ± 19	113 ± 10	0.277	0.536	0.484
Feeding behaviour during sow feeding times (%) on days 40–43							
Contacting sow feed	12.1 ± 1.0	13.0 ± 3.0	10.8 ± 1.9	11.7 ± 1.0	0.469	0.715	0.625
Contacting piglet feed ^7^	7.5 ± 0.8 ^y^	6.3 ± 1.7 ^xy^	6.6 ± 1.1 ^x^	7.1 ± 0.8 ^xy^	0.584	0.901	**0.071**
Contacting total feed	19.6 ± 1.2	19.2 ± 3.8	17.4 ± 2.2	18.7 ± 1.3	0.472	0.744	0.378
Feeding behaviour during the day (%) on day 41							
Contacting sow feed	4.4 ± 0.5	4.3 ± 1.5	5.0 ± 1.1	5.0 ± 0.6	0.733	**0.095**	0.944
Contacting piglet feed	4.7 ± 0.5	4.0 ± 1.0	4.3 ± 0.7	4.8 ± 0.5	0.875	0.391	0.550
Contacting total feed	9.0 ± 0.7	8.2 ± 1.9	9.1 ± 1.4	9.9 ± 0.8	0.723	0.402	0.827
Suckling behaviour: the presence at teats (no./7.5 h) on day 41							
Front and middle teats	4.7 ± 0.3	4.1 ± 0.6	4.3 ± 0.6	4.0 ± 0.4	0.679	0.512	0.826
Total teats	6.1 ± 0.2	6.4 ± 0.5	6.3 ± 0.4	5.8 ± 0.3	0.665	0.859	0.614
Alien teats	1.2 ± 0.2	2.0 ± 0.7	0.8 ± 0.3	1.8 ± 0.3	0.383	**0.010**	0.833
Skin lesions (no.)							
Day 27 ^8^	5.7 ± 0.7 ^b^	3.9 ± 1.2 ^a^	6.8 ± 1.3 ^ab^	4.5 ± 0.6 ^a^	0.667	**0.039**	**0.084**
Day 44	9.0 ± 0.7	7.1 ± 1.4	9.2 ± 1.4	7.5 ± 0.9	0.424	**0.047**	0.518

^1^ Focal piglets: On day 14 postpartum (p.p.), in each litter, the surviving second highest and lowest birthweight piglets from both sexes were selected as focal piglets. ^2^ Piglet type: Focal piglets were divided into four groups based on their birthweight (BiW) class and growth rate class. BiW class: high BiW (HBiW) focal piglets and low BiW (LBiW) focal piglets. Growth rate class (fast vs. slow): Within each BiW class, focal piglets were defined as fast-growing when their BW on day 44 p.p. was equal to or exceeded the median BW of their litter; other focal piglets were identified as slow growing. ^3^ *p*-values less than 0.100 are presented in bold. ^4^ The explanation of measured variables is further explained in the text. In a row, ^a,b^ values with different letters differ significantly (*p* < 0.05); ^x,y^ values with different letters tend to be different (0.05 < *p* < 0.10). ^5^ No statistics were performed for BiW. ^6^ The least squares means ± SEM of DM milk intake were 4.3 ± 0.1, 3.9 ± 0.3, 4.0 ± 0.2, 3.9 ± 0.2 for HBiW-fast, HBiW-slow, LBiW-fast, LBiW-slow piglets, respectively (the model was parameterized using a lognormal distribution). ^7^ The least squares means ± SEM of contacting piglet feed during sow feeding times (%) were −2.4 ± 0.1, −2.7 ± 0.2, −2.8 ± 0.2, −2.5 ± 0.1% for HBiW-fast, HBiW-slow, LBiW-fast, LBiW-slow, respectively (the data were parameterized of the beta distribution). ^8^ The least squares means ± SEM of skin lesions (no.) on day 27 were 1.7 ± 0.3, 1.2 ± 0.3, 1.4 ± 0.3, 1.4 ± 0.3 for HBiW-fast, HBiW-slow, LBiW-fast, LBiW-slow, respectively (the model was parameterized using a negative binomial distribution).

## Data Availability

The data were not deposited in an official repository. The datasets generated and analyzed during the current study are available from the corresponding author upon request.

## Supplementary Materials

The following supporting information can be downloaded at: https://www.mdpi.com/article/10.3390/ani13020297/s1, Figure S1: Number of dead and alive piglets in different birthweight (BiW) classes on days 0–26 p.p., days 27–44 p.p. up to day 44 of lactation in the multi-suckling system.

## References

[1] Moreira R.H.R., Pérez Palencia J.Y., Moita V.H.C., Caputo L.S.S., Saraiva A., Andretta I., Ferreira R.A., de Abreu M.L.T. (2020). Variability of piglet birth weights: A systematic review and meta-analysis. J. Anim. Physiol. Anim..

[2] Knol E.F., van der Spek D., Zak L.J. (2022). Genetic aspects of piglet survival and related traits: A review. J. Anim. Sci..

[3] Ward S.A., Kirkwood R.N., Plush K.J. (2020). Are larger litters a concern for piglet survival or an effectively manageable trait?. Animals.

[4] Paredes S.P., Jansman A.J.M., Verstegen M.W.A., Awati A., Buist W., Den Hartog L.A., Van Hees H.M.J., Quiniou N., Hendriks W.H., Gerrits W.J.J. (2012). Analysis of factors to predict piglet body weight at the end of the nursery phase. J. Anim. Sci..

[5] Douglas S.L., Edwards S.A., Kyriazakis I. (2014). Management strategies to improve the performance of low birth weight pigs to weaning and their long-term consequences. J. Anim. Sci..

[6] Van Nieuwamerongen S.E., Soede N.M., Van der Peet-Schwering C.M.C., Kemp B., Bolhuis J.E. (2017). Gradual weaning during an extended lactation period improves performance and behavior of pigs raised in a multi-suckling system. Appl. Anim. Behav. Sci..

[7] Thomsson O., Magnusson U., Bergqvist A.S., Eliasson-Selling L., Sjunnesson Y.C.B. (2018). Sow performance in multi-suckling pens with different management routines. Acta Vet. Scand..

[8] Lodge G.A., McDonald I. (1959). The relative influence of birth weight, milk consumption and supplementary food consumption upon the growth rates of suckling piglets. Anim. Sci..

[9] Thompson B.K., Fraser D. (1986). Variation in piglet weights: Development of within-litter variation over a 5-week lactation and effect of farrowing crate design. Can. J. Anim. Sci..

[10] Fraser D., Pajor E.A., Feddes J.J.R. (1994). The relationship between creep feeding behavior of piglets and adaptation to weaning: Effect of diet quality. Can. J. Anim. Sci..

[11] Muns R., Manzanilla E.G., Sol C., Manteca X., Gasa J. (2013). Piglet behavior as a measure of vitality and its influence on piglet survival and growth during lactation. J. Anim. Sci..

[12] Wiegert J.G., Garrison C., Knauer M.T. (2017). Characterization of birth weight and colostrum intake on piglet survival and piglet quality. J. Anim. Sci..

[13] Tang T., Gerrits W.J.J., Reimert I., van der Peet-Schwering C., Soede N.M. (2022). Variation in piglet body weight gain and feed intake during a 9-week lactation in a multi-suckling system. Animal.

[14] Milligan B.N., Fraser D., Kramer D.L. (2002). Within-litter birth weight variation in the domestic pig and its relation to pre-weaning survival, weight gain, and variation in weaning weights. Livest. Prod. Sci..

[15] Quiniou N., Dagorn J., Gaudré D. (2002). Variation of piglets’ birth weight and consequences on subsequent performance. Livest. Prod. Sci..

[16] Hales J., Moustsen V.A., Nielsen M.B.F., Hansen C.F. (2013). Individual physical characteristics of neonatal piglets affect preweaning survival of piglets born in a noncrated system. J. Anim. Sci..

[17] Van Nieuwamerongen S.E., Soede N.M., Van der Peet-Schwering C.M.C., Kemp B., Bolhuis J.E. (2015). Development of piglets raised in a new multi-litter housing system vs. conventional single-litter housing until 9 weeks of age. J. Anim. Sci..

[18] Rehfeldt C., Kuhn G. (2006). Consequences of birth weight for postnatal growth performance and carcass quality in pigs as related to myogenesis. J. Anim. Sci..

[19] Beaulieu A.D., Aalhus J.L., Williams N.H., Patience J.F. (2010). Impact of piglet birth weight, birth order, and litter size on subsequent growth performance, carcass quality, muscle composition, and eating quality of pork. J. Anim. Sci..

[20] Douglas S.L., Edwards S.A., Sutcliffe E., Knap P.W., Kyriazakis I. (2013). Identification of risk factors associated with poor lifetime growth performance in pigs. J. Anim. Sci..

[21] Madsen J.G., Bee G. (2015). Compensatory growth feeding strategy does not overcome negative effects on growth and carcass composition of low birth weight pigs. Animal.

[22] Douglas S.L., Edwards S.A., Kyriazakis I. (2016). Are all piglets born lightweight alike? Morphological measurements as predictors of postnatal performance. J. Anim. Sci..

[23] Tang T., Gerrits W.J.J., Soede N.M., van der Peet-Schwering C.M.C., Reimert I. (2023). Effects of timing of grouping and split-weaning on growth performance and behaviour of piglets in a multi-suckling system. Appl. Anim. Behav. Sci..

[24] Weary D.M., Pajor E.A., Bonenfant M., Fraser D., Kramer D.L. (2002). Alternative housing for sows and litters: Part 4. Effects of sow-controlled housing combined with a communal piglet area on pre-and post-weaning behaviour and performance. Appl. Anim. Behav. Sci..

[25] Turner S.P., Farnworth M.J., White I.M., Brotherstone S., Mendl M., Knap P., Penny P., Lawrence A.B. (2006). The accumulation of skin lesions and their use as a predictor of individual aggressiveness in pigs. Appl. Anim. Behav. Sci..

[26] Tang T., van der Peet-Schwering C.M.C., Soede N.M., Laurenssen B.F.A., Bruininx E.M.A.M., Bos E.J., Gerrits W.J.J. (2022). A dual marker technique to estimate individual feed intake in young pigs. Animal.

[27] Smit H.J., Taweel H.Z., Tas B.M., Tamminga S., Elgersma A. (2005). Comparison of techniques for estimating herbage intake of grazing dairy cows. J. Dairy Sci..

[28] Theil P.K., Nielsen T.T., Kristensen N.B., Labouriau R., Danielsen V., Lauridsen C., Jakobsen K. (2002). Estimation of milk production in lactating sows by determination of deuterated water turnover in three piglets per litter. Acta Agric. Scand. A Anim. Sci..

[29] Hurley W.L., Farmer C. (2015). Composition of sow colostrum and milk. The Gestating and Lactating Sow.

[30] Stroup W.W., Milliken G.A., Claassen E.A., Wolfinger R.D. (2018). SAS for Mixed Models: Introduction and Basic Applications.

[31] Thomsson O., Sjunnesson Y., Magnusson U., Eliasson-Selling L., Wallenbeck A., Bergqvist A.S. (2016). Consequences for piglet performance of group housing lactating sows at one, two, or three weeks post-farrowing. PLoS ONE.

[32] Kirkden R.D., Broom D.M., Andersen I.L. (2013). Invited review: Piglet mortality: Management solutions. J. Anim. Sci..

[33] Heuß E.M., Pröll-Cornelissen M.J., Neuhoff C., Tholen E., Große-Brinkhaus C. (2019). Invited review: Piglet survival: Benefits of the immunocompetence. Animal.

[34] Vande Pol K.D., Bautista R.O., Harper H., Shull C.M., Brown C.B., Ellis M. (2021). Effect of within-litter birth weight variation after cross-fostering on piglet preweaning growth and mortality. Transl. Anim. Sci..

[35] Verdon M., Morrison R.S., Rault J.L. (2020). The welfare and productivity of sows and piglets in group lactation from 7, 10, or 14 d postpartum. J. Anim. Sci..

[36] Marchant J.N., Rudd A.R., Mendl M.T., Broom D.M., Meredith M.J., Corning S., Simmins P.H. (2000). Timing and causes of piglet mortality in alternative and conventional farrowing systems. Vet. Rec..

[37] Pedersen L.J., Berg P., Jørgensen G., Andersen I.L. (2011). Neonatal piglet traits of importance for survival in crates and indoor pens. J. Anim. Sci..

[38] Herpin P., Vincent A., Damon M. (2004). Effect of breed and body weight on thermoregulatory abilities of European (Pie-train×(Landrace× Large White)) and Chinese (Meishan) piglets at birth. Livest. Prod. Sci..

[39] Kammersgaard T.S., Pedersen L.J., Jørgensen E. (2011). Hypothermia in neonatal piglets: Interactions and causes of individual differences. J. Anim. Sci..

[40] Vanden Hole C., Aerts P., Prims S., Ayuso M., Van Cruchten S., Van Ginneken C. (2018). Does intrauterine crowding affect locomotor development? A comparative study of motor performance, neuromotor maturation and gait variability among piglets that differ in birth weight and vitality. PLoS ONE.

[41] Herpin P., Damon M., Le Dividich J. (2002). Development of thermoregulation and neonatal survival in pigs. Livest. Prod. Sci..

[42] Weary D.M., Pajor E.A., Thompson B.K., Fraser D. (1996). Risky behaviour by piglets: A trade off between feeding and risk of mortality by maternal crushing?. Anim. Behav..

[43] Fraser D. (1990). Behavioural perspectives on piglet survival. J. Reprod. Fertil. Suppl..

[44] Andersen I.L., Nævdal E., Bøe K.E. (2011). Maternal investment, sibling competition, and offspring survival with increasing litter size and parity in pigs (*Sus scrofa*). Behav. Ecol. Sociobiol..

[45] Oostindjer M., Bolhuis J.E., Mendl M., Held S., van den Brand H., Kemp B. (2011). Learning how to eat like a pig: Effectiveness of mechanisms for vertical social learning in piglets. Anim. Behav..

[46] Van der Peet-Schwering C.M.C., Troquet L.M.P., Binnendijk G.P., Knol E. (2013). Effect of Genetic Background and Birth Weight on Performance of Piglets and Growing and Finishing Pigs.

[47] Alvarenga A.L.N., Chiarini-Garcia H., Cardeal P.C., Moreira L.P., Foxcroft G.R., Fontes D.O., Almeida F.R.C.L. (2013). Intra-uterine growth retardation affects birthweight and postnatal development in pigs, impairing muscle accretion, duodenal mucosa morphology and carcass traits. Reprod. Fertil. Dev..

[48] Van der Peet-Schwering C.M.C., Verschuren L.M., Bergsma R., Hedemann M.S., Binnendijk G.P., Jansman A.J. (2021). The effects of birth weight and estimated breeding value for protein deposition on nitrogen efficiency in growing pigs. J. Anim. Sci..

[49] Michiels J., De Vos M., Missotten J., Ovyn A., De Smet S., Van Ginneken C. (2013). Maturation of digestive function is retarded and plasma antioxidant capacity lowered in fully weaned low birth weight piglets. Br. J. Nutr..

[50] Gill J.C., Thomson W. (1956). Observations on the behaviour of suckling pigs. Brit. J. Anim. Behav..

[51] Fraser D., Nicholls C., Fagan W. (1985). A sow milking machine designed to compare the yield of different teats. J. Agric. Eng. Res..

[52] Skok J., Brus M., Škorjanc D. (2007). Growth of piglets in relation to milk intake and anatomical location of mammary glands. Acta Agric. Scand. A..

[53] Ogawa S., Tsukahara T., Tsuruta T., Nishibayashi R., Okutani M., Nakatani M., Higashide K., Iida S., Nakanishi N., Ushida K. (2014). The evaluation of secretion volume and immunoglobulin A and G concentrations in sow colostrum from anterior to posterior teats. Anim. Sci. J..

[54] Hurley W.L. (2001). Mammary gland growth in the lactating sow. Livest. Prod. Sci..

[55] Hartsock T.G., Graves H.B., Baumgardt B.R. (1977). Agonistic behavior and the nursing order in suckling piglets: Relationships with survival, growth and body composition. J. Anim. Sci..

[56] Farmer C. (2019). Mammary development in lactating sows: The importance of suckling. Animal.

[57] Guevara R.D., Pastor J.J., Manteca X., Tedo G., Llonch P. (2022). Systematic review of animal-based indicators to measure thermal, social, and immune-related stress in pigs. PLoS ONE.

[58] Van Kerschaver C., Vandaele M., Degroote J., Van Tichelen K., Fremaut D., Van Ginneken C., Michiels J. (2021). Effect of starting time of co-mingling non-littermates during lactation on performance and skin lesions of sows and piglets. Livest. Sci..

[59] Bernardino T., Tatemoto P., Morrone B., Mazza Rodrigues P.H., Zanella A.J. (2016). Piglets born from sows fed high fibre diets during pregnancy are less aggressive prior to weaning. PLoS ONE.

[60] Paredes S.P., Jansman A.J.M., Verstegen M.W.A., Den Hartog L.A., Van Hees H.M.J., Bolhuis J.E., Van Kempen T.A.T.G., Gerrits W.J.J. (2014). Identifying the limitations for growth in low performing piglets from birth until 10 weeks of age. Animal.

[61] Hoy S., Schamun S., Weirich C. (2012). Investigations on feed intake and social behaviour of fattening pigs fed at an electronic feeding station. Appl. Anim. Behav. Sci..

[62] Nielsen B.L., De Jong I.C., Vries T.J.D., Phillips C.J.C. (2016). The use of feeding behaviour in the assessment of animal welfare. Nutrition and the Welfare of Farm Animals.

[63] Valros A., Sali V., Hälli O., Saari S., Heinonen M. (2021). Does weight matter? Exploring links between birth weight, growth and pig-directed manipulative behaviour in growing-finishing pigs. Appl. Anim. Behav. Sci..

[64] Algers B., Jensen P., Steinwall L. (1990). Behaviour and weight changes at weaning and regrouping of pigs in relation to teat quality. Appl. Anim. Behav. Sci..

[65] Yang C.H., Ko H.L., Salazar L.C., Llonch L., Manteca X., Camerlink I., Llonch P. (2018). Pre-weaning environmental enrichment increases piglets’ object play behaviour on a large scale commercial pig farm. Appl. Anim. Behav. Sci..

[66] Bus J.D., Boumans I.J., Webb L.E., Bokkers E.A. (2021). The potential of feeding patterns to assess generic welfare in growing-finishing pigs. Appl. Anim. Behav. Sci..

[67] Olsen A.N.W., Dybkjaer L., Vestergaard K.S. (1998). Cross-suckling and associated behaviour in piglets and sows. Appl. Anim. Behav. Sci..

[68] Quesnel H., Farmer C., Theil P.K., Farmer C. (2015). Colostrum and milk production. The Gestating and Lactating Sow.

[69] Berkeveld M., Langendijk P., van Beers-Schreurs H.M., Koets A.P., Taverne M.A., Verheijden J.H. (2007). Postweaning growth check in pigs is markedly reduced by intermittent suckling and extended lactation. J. Anim. Sci..

