# Supplementary material for: Effects of Birthweight of Piglets in a Multi-Suckling System on Mortality, Growth Rate, Catch-Up Growth, Feed Intake and Behaviour
Source: Animals (Basel). 2023 Jan 14;13(2):297. doi: 10.3390/ani13020297 (PMC9855195; doi:10.3390/ani13020297)
Supplement: Supplementary file 1 [file animals-13-00297-s001.zip › animals-2123208-supplementary.pdf]

Effects of Birthweight of Piglets in a Multi-Suckling System on Mortality, Growth Rate, Catch-Up Growth, Feed Intake and Behaviour

Tianyue Tang, Walter J.J. Gerrits, Carola M.C. van der Peet-Schwering, Nicoline M. Soede and Inonge Reimert

Animals journal

Supplementary material

Supplementary Figure S1

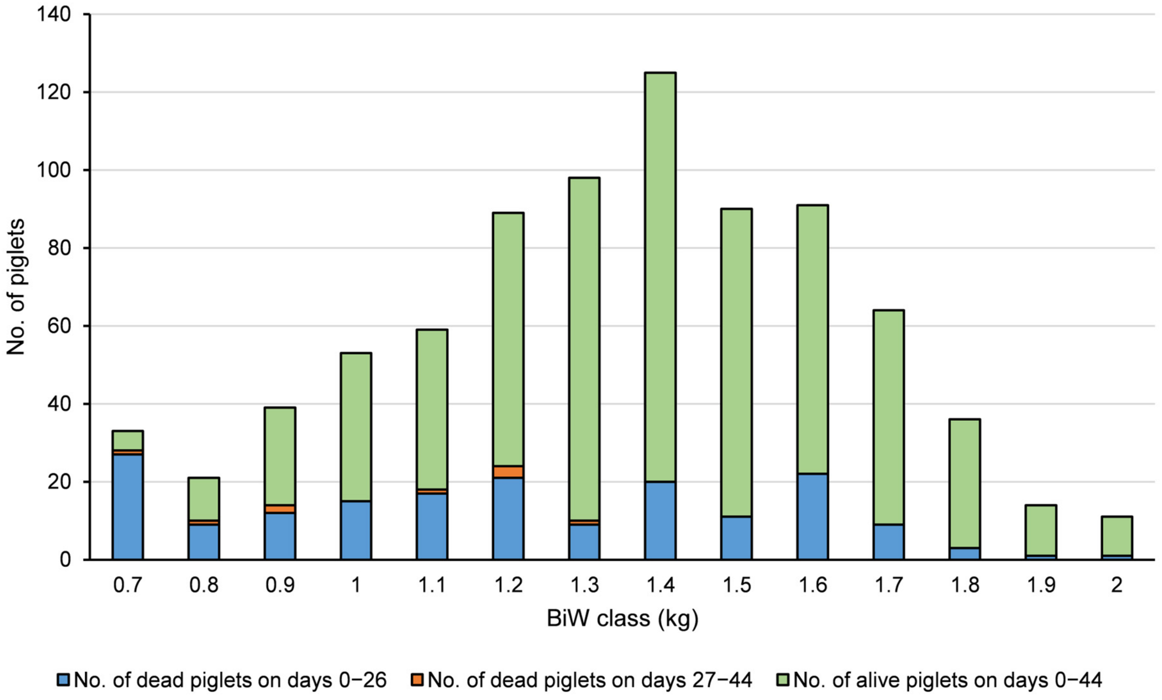

Figure S1. Number of dead and alive piglets in different birthweight (BiW) classes on days 0–26 p.p., days 27–44 p.p. up to day 44 of lactation in the multi-suckling system. Piglets with BiW <0.7 and >2.0 kg are included in the 0.7 and 2.0 kg BiW class, respectively. Stillborn piglets were excluded from the data set and piglets which were cross-fostered in the MS system were included in the data set. Data were merged from two animal experiments conducted in the MS system. In exp 1, piglets were grouped on days 8–9 p.p. In exp 2, piglets in control groups were grouped on days 8–9 p.p. and no split-weaning was applied; piglets in treatment groups were grouped on days 13–14 p.p. and the three heaviest non-focal piglets per litter were split-weaned on day 35 p.p. Previously we found no treatment effect on most of the measured variables in exp 2.
